# Arecoline Triggers Psychostimulant Responses by Modulating the Intestinal Microbiota to Influence Neurotransmitter Levels and Digestive Enzyme Activity

**DOI:** 10.3390/ph18060794

**Published:** 2025-05-25

**Authors:** Jiaxin Di, Shiqin Xie, Junxi Shen, Leyao Fang, Zhoujin Tan, Xuejuan Liang

**Affiliations:** 1 School of Pharmacy, Hunan University of Chinese Medicine, Changsha 410208, China; 18216375395@163.com; 2 School of Traditional Chinese Medicine, Hunan University of Chinese Medicine, Changsha 410208, China; beibingyangfacaileme@outlook.com (S.X.); shenjunxi816@163.com (J.S.); 18229704061@163.com (L.F.); 3 Institute of Innovative Traditional Chinese Medicine, Hunan Academy of Chinese Medicine, Changsha 410013, China

**Keywords:** *Areca catechu* L., arecoline, neurotransmitters, intestinal microbiota, enzyme activity

## Abstract

**Background:** *Areca catechu* L. is an evergreen tree belonging to the Arecaceae family. As an important traditional Chinese medicine, it has wide applications in the field of herbal medicine. Arecoline is the main active component responsible for its medicinal effects and plays a key role in its central nervous system (CNS) stimulant properties. **Methods:** This study investigated the excitatory effects of arecoline by analyzing behavioral changes in mice, neurotransmitter levels, the intestinal microbiota composition, and enzymatic activities. We further explored the bidirectional interactions between the intestinal microbial ecosystem and the nervous system following arecoline exposure. **Results:** Arecoline administration significantly increased the activity time ratio in mice (*p* < 0.05). It also elevated fecal lactase and amylase activities (*p* < 0.05), suggesting enhanced carbohydrate metabolism that may be one of the reasons for the increased activity time of mice. Serum analysis showed decreased 5-hydroxytryptamine (5-HT, *p* < 0.05), increased dopamine (DA) and brain-derived neurotrophic factor (BDNF) levels (*p* < 0.001), and no significant change in γ-aminobutyric acid (GABA). These findings suggest that arecoline may also play a role in modulating neurotransmitter balance. At the genus level, *Escherichia* was significantly enriched and positively correlated with DA, BDNF, and GABA, while *Clostridium* abundance decreased and was positively correlated with 5-HT. **Conclusions:** Arecoline administration altered multiple enzymatic activities and the microbial composition abundance in the mouse intestine, eliciting psychostimulant effects while maintaining neurotransmitter homeostasis. This study provides an experimental foundation for further pharmacological exploitation of arecoline.

## 1. Introduction

The seeds of *Areca catechu* L. (betel nut) are economically important crop in tropical and subtropical regions that is widely cultivated in southern China. Betel nut has a long history of being used to refresh the mind. Despite its extensive use, public concerns regarding its adverse health effects have overshadowed its potential medicinal benefits. As one of the ‘Four Major Southern Medicinal Plants’ (*Areca catechu* L., *Amomum villosum* Lour., *Alpinia oxyphylla* Miq., and *Morinda officinalis* F. C. How.), both its seeds and fruits have been extensively utilized in traditional Chinese medicine (TCM). With the development of science and technology, many bioactive components in betel nut have gradually entered people’s field of vision. Researchers have isolated arecoline, polysaccharides, saponins, and polyphenols from betel nut, elucidating their pharmacological potential [1,2,3]. As a principal natural active ingredient in arecae semen extract, arecine has positive antioxidant and anti-inflammatory properties [4]. Arecoline has also been shown to increase glycolysis and reduce the mRNA expression of pH-regulated proteins, leading to the development of anticancer therapeutics [5].

Furthermore, arecoline exerts significant neuromodulatory and gastroenterological effects. As a naturally occurring psychoactive alkaloid, arecoline is an acetylcholine receptor agonist that targets both nicotinic (nAChRs) and muscarinic (mAChRs) receptors. On the one hand, after arecoline activates M and N receptors, arecoline stimulates the central nervous system, producing multiple psychoactive effects, including euphoria, analgesia, fatigue resistance, and anxiolysis [6]. Studies evaluating arecoline’s anti-fatigue effects have demonstrated its ability to promote neurotransmitter release, significantly increasing the levels of dopamine (DA), 5-hydroxytryptamine (5-HT), and γ-aminobutyric acid (GABA) in fatigued mice, thereby exerting anti-fatigue effects [7]. The anterior cingulate cortex (ACC) plays a pivotal role in modulating chronic pain and associated anxiety- and depression-like behaviors. Arecoline can inhibit ACC transmission by inhibiting certain underlying neurotransmissions, resulting in analgesic and antidepressant effects [8]. Furthermore, brain-derived neurotrophic factor (BDNF), a critical regulator of neurological function, plays pivotal roles in both neuronal growth and synaptic neurotransmission [9]. Studies have shown that surgery-induced neurological disorders can be ameliorated by restoring BDNF expression in vivo [10], so changes in BDNF levels in vivo can also respond to nervous system excitability to some extent. On the other hand, at specific dosages, arecoline acts as an M receptor agonist, increasing gastrointestinal smooth muscle tone and enhancing peristaltic motility. Studies have demonstrated that dietary supplementation with appropriate doses of arecoline in grass carp (*Ctenopharyngodon idella*) enhances intestinal structural integrity while increasing the activity of key digestive enzymes, including hepatopancreatic trypsin, chymotrypsin, lipase, and amylase. Concurrent elevation of brush border enzyme activity further promotes gastrointestinal motility and enhances nutrient transport [11].

The intestinal microbiota, as the ‘second genome’ of the human body, is colonized by tens of thousands of microorganisms, including bacteria, fungi, and viruses [12]. With rapid advances in modern intestinal microbiome technologies, the critical linkage between microbial homeostasis and human health has become increasingly evident, and intestinal microbiota homeostasis has become an important intervention target for disease treatment. When this stable state balance is maintained, the body produces protective immunity. Once this stable state is disrupted, it may lead to compromised barrier function, inflammatory responses, and immune dysfunction. A study revealed significant correlations between the content of neurotransmitters such as 5-HT, GABA, and norepinephrine (NE) in mice after the administration of arecoline and the abundance of specific intestinal microbiota, including *Bacteroides faecichinchillae*, *Bacteroides vulgatus*, *Muribaculum intestinale*, and *Mucispirillum schaedleri.* Arecoline has a beneficial effect on the human nervous system by regulating intestinal microbes and further influencing the secretion of neurotransmitters [13]. However, arecoline can also regulate the production of serum metabolites by altering the abundance and composition of the mouse intestinal flora, thereby affecting the occurrence and development of acute ulcerative colitis [14]. Arecoline has various pharmacological effects and has great potential as a drug for treating various diseases. Further research is still needed in the future to reduce or eliminate its toxicity to develop it into a new drug [15]. Our team’s previous research on the stimulant and acute toxicity of arecoline revealed that arecoline has a significant effect on countering the hypnotic effect of sodium phenobarbital and can cause excitement in mice; the stimulant effect increases with increasing dose. However, arecoline displayed moderate toxicity, potentially inducing hepatotoxicity and damage to other visceral organs [16]. Further investigations revealed that oral administration of arecoline at 2 mg/(kg·d) elicited stimulatory effects in mice, and the damage to the brain and liver was relatively mild at this dose [17]. However, the precise mechanisms underlying the systemic effects of arecoline, particularly the role of the intestinal microbiota in this process. How the intestinal flora affects the central nervous system through the regulation of neurotransmitters after the action of arecaine, exerting an anti-fatigue effect. To gain a deeper understanding of the mechanism of action of arecoline in the body, this study starts from the perspective of the intestinal flora to explore the effects of arecoline on the mouse nervous system. This study aims to enhance our understanding of the stimulatory effects of arecoline, further strengthen our understanding of the complex relationship between the brain and the intestines, provide both an experimental basis for the future development and utilization of arecoline, and offer new ideas and methods for the treatment of many diseases, such as degenerative diseases.

## 2. Results

### 2.1. Effects of Arecoline on Mental State and Spontaneous Activity in Mice

**Figure 1:**
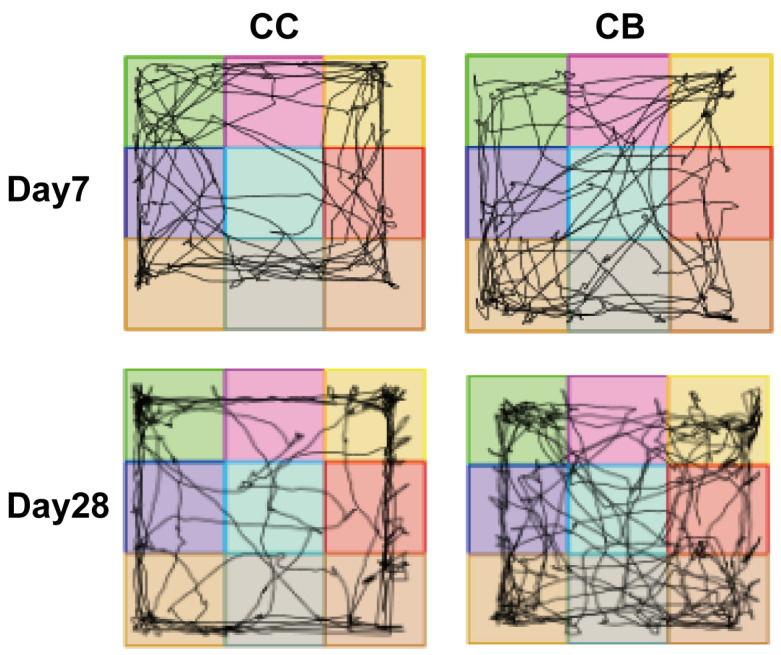
Open field experimental activity trajectory map.

The open field test was used to evaluate the mental state and spontaneous activity of the mice, with assessments conducted on days 7, 14, 21, and 28 of the experiment. As shown in Table 1, compared with the normal group, the arecoline group presented an increased activity time ratio and center zone duration on days 7, 14, and 21, although these differences were not statistically significant (*p* > 0.05). On day 28, compared with the normal group, the arecoline group presented a significant increase in the activity-time ratio (*p* < 0.05), with trends toward increased time spent in the center zone and center zone distance traveled, although these trends did not reach statistical significance (*p* > 0.05). Figure 1 further illustrates that on day 28, the arecoline group of mice showed more concentrated movement trajectories in the center zone and markedly increased activity frequency than the normal group did.

### 2.2. Effects of Arecoline on Fecal Enzyme Activity in Mice

**Figure 2:**
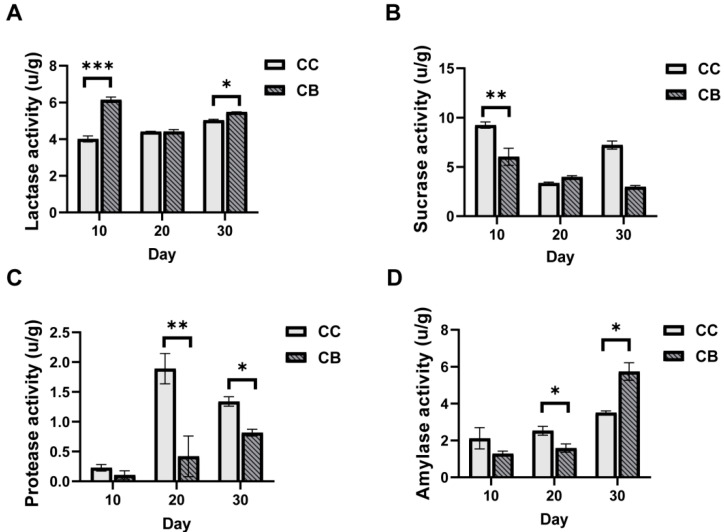
Effects of arecoline on fecal enzyme activity in mice: (**A**): lactase activity; (**B**): sucrase activity; (**C**): protease activity; (**D**): amylase activity. Values are expressed as mean ± standard deviation (n = 3). * *p* < 0.05, ** *p* < 0.01, *** *p* < 0.001.

Changes in digestive enzyme activities in mice can reflect the effects of arecoline on the gastrointestinal system to some extent. As shown in Figure 2A–D, on day 10 of arecoline administration, compared with the normal group, mice treated with arecoline showed significantly increased lactase activity (*p* < 0.001) and significantly decreased sucrase activity (*p* < 0.01) in intestinal contents, while protease and amylase activities showed no significant changes. On day 20 of arecoline administration, lactase and sucrase activities in intestinal contents showed no significant differences compared with the normal group, while protease and amylase activities decreased significantly (*p* < 0.01, *p* < 0.05). On the final experimental day, compared with the normal group, arecoline significantly increased lactase and amylase activities (*p* < 0.05, *p* < 0.05), while the activities of sucrase and protease decreased to a certain extent (*p* > 0.05, *p* < 0.05).

### 2.3. Effects of Arecoline on Changes in Serum GABA, DA, 5-HT and BDNF Levels in Mice

**Figure 3:**
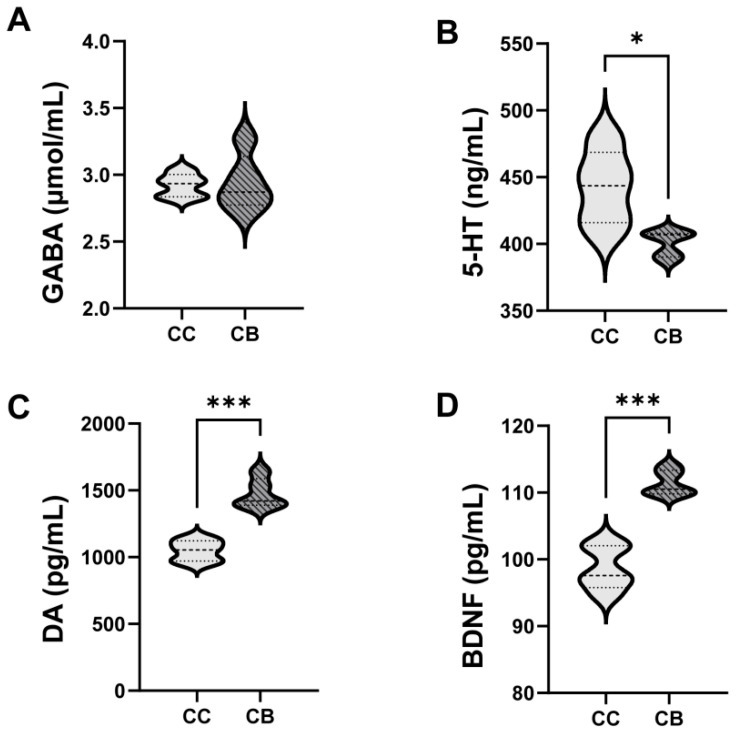
Effects of arecoline on GABA, BDNF, DA, and 5-HT in mice. Values are expressed as mean ± standard deviation (n = 5). (**A**): GABA; (**B**): 5-HT; (**C**): DA; (**D**): BDNF. * *p* < 0.05, *** *p* < 0.001.

After arecoline administration, the serum GABA levels in the mice tended to decrease, but there was no significant difference compared with those in the normal group (*p* > 0.05; Figure 3A). Serum 5-HT levels were significantly lower than those in the normal group (*p* < 0.05; Figure 3B). In addition, arecoline administration significantly increased the serum DA and BDNF levels in the mice (*p* < 0.01; Figure 3C,D).

### 2.4. Effects of Arecoline on the Intestinal Microbiota Composition in Mice

#### 2.4.1. Quality Assessment of Sequencing Data

**Figure 4:**
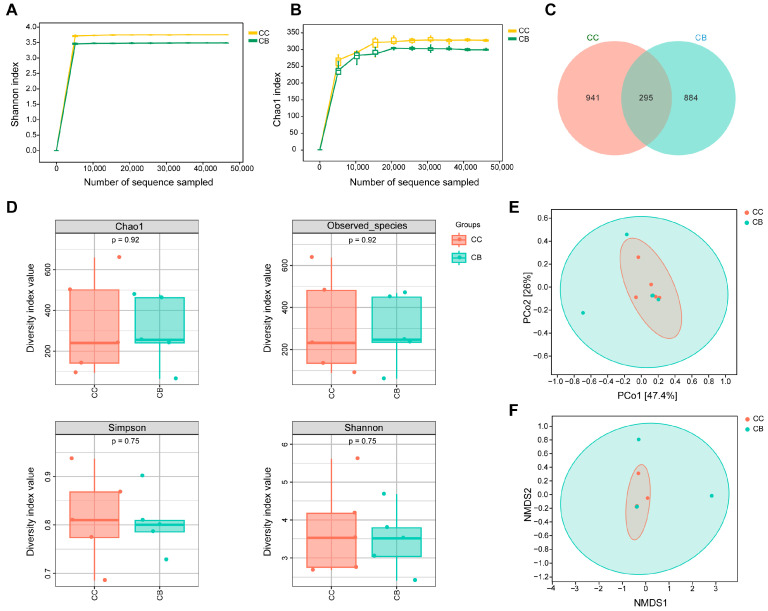
Effects of arecoline on the microbial abundance and diversity of intestinal contents in mice. (**A**): Shannon rarefaction curve; (**B**): Chao1 rarefaction curve; (**C**): Venn diagram (ASV level); (**D**): Alpha diversity indices (Chao1 index, Observed_species index, Simpson index, Shannon index); (**E**): principal coordinate analysis (PCoA); (**F**): nonmetric multidimensional scaling (NMDS).

Rarefaction curves can be used to evaluate whether sequencing quality meets standards and, to some extent, reflect species diversity within samples. Moreover, the impact of sequencing depth on the diversity of observed samples can be reflected by the degree of flatness of the curve. As shown in Figure 4A,B, as the sequencing depth increased, the Shannon index curve and Chao1 index curve no longer showed significant rises and instead plateaued. This finding indicates that the sequencing depth was sufficient to cover all the species present in the samples, indicating that the sequencing data met the research requirements and were reliable.

#### 2.4.2. Effects of Arecoline on Microbial Abundance and Diversity of Intestinal Contents in Mice

As shown in Figure 4C, the Venn diagram reveals 295 shared ASVs across groups, with the normal group containing 941 unique ASVs and the arecoline-treated group containing 884 unique ASVs.

Alpha diversity reflects the richness and diversity of microbial communities within samples, where the Chao1 index and Observed_species index assess species richness, and the Shannon index and Simpson index evaluate species diversity. As shown in Figure 4D, the Chao1 index and Observed_species index of the arecoline group mice both showed a slight increase, while the Shannon index and Simpson index both showed a slight decrease, but there was no statistically significant difference between the groups (*p* > 0.05).

The differences in species composition between sample groups can be displayed through beta diversity. Unconstrained ordination methods, including principal coordinate analysis (PCoA) and nonmetric multidimensional scaling (NMDS), were employed to reduce the dimensionality of multivariate microbial data. The distance of each group’s projection from the graph reflects the similarity of community composition among the groups in the same dimension. As illustrated in Figure 4E,F, compared to the normal group, arecoline administration increased microbial species diversity while also enhancing inter-individual variability in intestinal microbiota composition.

#### 2.4.3. Effects of Arecoline on the Dominant Microbiota of Intestinal Contents in Mice

**Figure 5:**
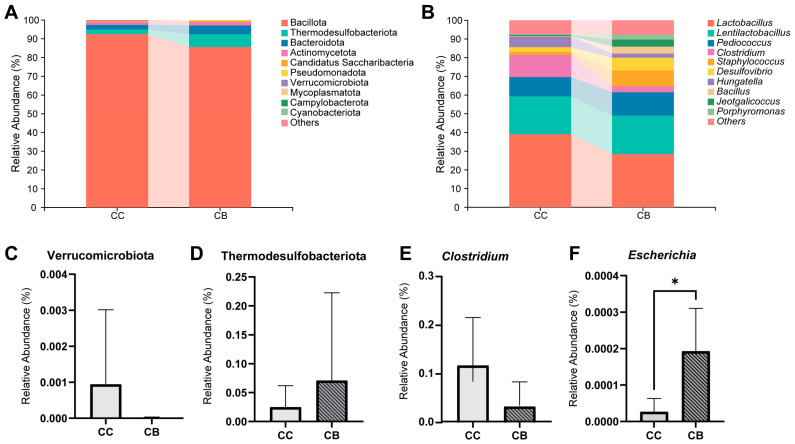
Effect of arecoline on the dominant microbiota of the intestinal contents of mice: (**A**): relative abundance at the phylum level; (**B**): relative abundance at the genus level; (**C**,**D**): differential phyla; (**E**,**F**): differential genera. * *p* < 0.05.

To investigate the effects of arecaine on the composition of small intestinal contents in mice, we performed statistical analyses of the top 10 species in each group at the phylum and genus levels and presented the results in histograms. As shown in Figure 5A, Bacillota was identified as the most dominant phylum in both groups, followed by Thermodesulfobacteriota and Bacteroidota as the second and third most abundant phyla, respectively. Arecoline administration resulted in a 0.09% decrease in the relative abundance of Verrucomicrobiota and a 4.6% increase in Thermodesulfobacteriota (Figure 5C,D). Figure 5B shows the change in the abundance of the dominant flora in each group at the genus level, with *Lactobacillus* being the first dominant genus. As shown in Figure 4E,F, arecoline reduced the relative abundance of *Clostridium* in the intestinal contents of the mice by 8.45% and significantly increased the relative abundance of *Escherichia* compared with those in the normal group (*p* < 0.05).

#### 2.4.4. Effects of Arecoline on Characteristic Microbiota of Intestinal Contents in Mice

**Figure 6:**
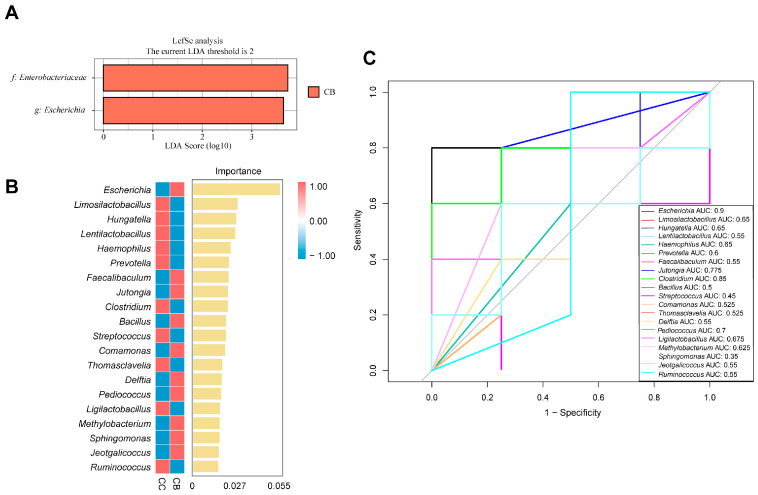
Effect of arecoline on the characteristic microbiota of the intestinal contents if mice: (**A**): bar plot of differentially abundant species (LDA ≥ 2); (**B**): genus-level random forest analysis; (**C**): genus-level ROC analysis (CC vs. CB).

LEfSe analysis was performed to identify differentially abundant taxa across all classification levels, with significantly enriched species in each group displayed via LDA score distribution histograms (LDA ≥ 2). As shown in Figure 6A, *Escherichia* was significantly enriched in the arecoline group, representing a characteristic genus of this group.

To further identify key species distinguishing between the normal and arecoline groups, we constructed a random forest model and selected the top 20 characteristic genus-level bacteria for each group (Figure 6B). Subsequent ROC analysis of these selected bacteria, using an AUC > 0.8 threshold, validated their diagnostic accuracy for intergroup differentiation. Figure 6C shows that *Escherichia* (AUC = 0.9) and *Clostridium* (AUC = 0.85) served as characteristic genera for the normal and arecoline groups, respectively.

#### 2.4.5. Correlation Analysis

**Figure 7:**
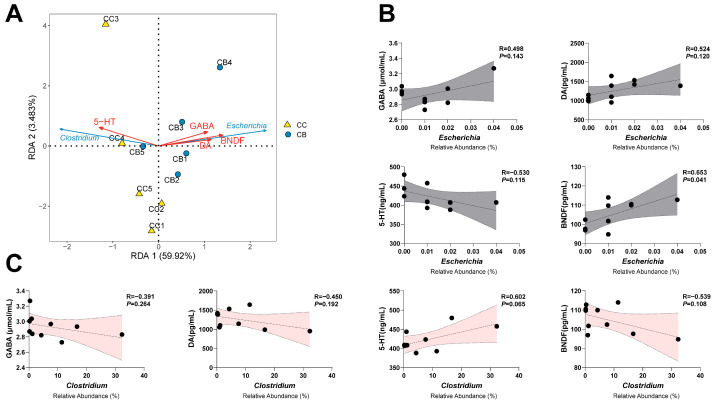
Correlation analysis between the intestinal microbiota and neurotransmitters: (**A**): redundancy analysis (RDA), (**B**): scatter plots of *Escherichia* with GABA, DA, 5-HT and BDNF; (**C**): scatter plots of *Clostridium* with GABA, DA, 5-HT and BDNF.

We investigated the correlations between the serum levels of GABA, DA, 5-HT, and BDNF and the characteristic bacteria *Escherichia* and *Clostridium* via RDA and correlation analysis. As shown in Figure 7A–C, 5-HT levels were positively correlated with *Clostridium* abundance and negatively correlated with *Escherichia* abundance; GABA, DA, and BDNF levels were positively correlated with *Escherichia* abundance and negatively correlated with *Clostridium* abundance; notably, BDNF was significantly positively correlated with *Escherichia* abundance (*p* < 0.05).

## 3. Discussion

### 3.1. Arecoline May Exert Its Stimulatory Effects by Modulating Neurotransmitter Activity

Betel nut is recognized as one of the four major addictive substances alongside caffeine, nicotine, and alcohol. Its primary active component, arecoline, binds to relevant receptors and induces a series of effects: stimulating the nervous system, enhancing neural excitability, and producing antidepressant and analgesic effects. However, prolonged use or uncontrolled dosage may lead to addiction and even carcinogenic consequences [18,19]. To evaluate neurological conditions such as anxiety and depression in animals, researchers commonly employ the open field test to assess spontaneous activity and exploratory behavior. It can be reflected by indicators such as a mouse’s activity time, the time spent in the central zone, and the total distance traveled [20]. In the open field test, mice treated with arecoline exhibited higher activity levels compared to the normal group. On day 28 of the experiment, compared with the normal group, a significant increase in the activity time was observed in the arecoline group. This shows that arecoline to a certain extent enhances the exploratory behavior and spontaneous activity of mice, which can be considered an obvious manifestation of the stimulant effect of arecoline.

Further analysis of neurotransmitter levels revealed that compared with the normal group arecoline significantly decreased serum 5-HT levels (*p* < 0.05) while markedly increasing DA levels (*p* < 0.001) in the serum. 5-HT, synthesized from tryptophan through multiple biosynthetic steps, is a multifunctional neurotransmitter that exerts critical regulatory effects on both the nervous system and gastrointestinal tract [21,22,23]. Research has shown that sea buckthorn seed oil can reduce hypothalamic 5-HT levels in mice, counteract swimming-induced fatigue, and exert excellent anti-fatigue effects [24]. Furthermore, in the gastrointestinal system, serum 5-HT levels are negatively correlated with the contents of short-chain fatty acid salts (butyrate and acetate) in mouse feces, which may impair normal gastrointestinal function [25]. DA, along with epinephrine and norepinephrine, belongs to the catecholamine family and is closely associated with mental-emotional activities, gastrointestinal motility, pituitary secretion, and cardiovascular function [26]. Concurrently, studies have demonstrated that arecoline can stabilize the central neurotransmitter balance by suppressing the increase in the W_5-HT_/W_DA_ ratio [27]. These findings align with the observed trends of 5-HT and DA level alterations in this study. Arecoline reduces serum 5-HT levels, thereby attenuating excessive neural excitation while simultaneously modulating serum DA concentrations to maintain interneuronal equilibrium. This mechanism prevents various adverse effects associated with neurotransmitter imbalance, including anxiety, depression, and more severe psychiatric disorders. This mechanism helps stabilize the nervous system and may also be one of the ways by which arecoline stimulates or regulates the mental state. Furthermore, our experimental results revealed that arecoline significantly increased serum BDNF levels in mice (*p* < 0.001). As the most abundant neurotrophic factor in the body, BDNF plays crucial roles in maintaining and promoting the development, differentiation, growth, and regeneration of various neurons and is widely expressed in the central nervous system, intestinal, and other tissues [28]. Studies have revealed that astrocytes, which play a key role in the central nervous system, can specifically detect extracellular BDNF and activate intracellular responses, thereby influencing specific signaling pathways that modulate neuronal excitability [29]. Consequently, as BDNF is a “nutrient source” for neurons, elevated BDNF levels provide robust support for neuronal excitation, establishing a solid foundation for the stimulatory effects of arecoline. GABA, a biologically active amino acid crucial for cerebral energy metabolism, plays significant roles in antidepressant effects, antioxidant activity, anti-inflammatory responses, and intestinal protection [30]. In this study, we observed no statistically significant alterations in the serum GABA levels, indicating that the administration of coline at this dosage and duration had no measurable influence on the circulating GABA concentrations. Nevertheless, it should be noted that physiological GABA levels interact with multiple neural receptors; thus, their functional mechanisms are modulated by various factors.

The regulatory effect of arecoline on neurotransmitters showed different intensities in different sites, and combined with the results of previous studies, we found that the changes of neurotransmitters in the liver and brain tissues of mice were not as significant as in serum after the use of arecaine [17]. This may be because orally administered drugs are first absorbed through the gastrointestinal tract, then metabolized by the liver before entering systemic circulation [31,32]. The liver’s strong metabolic capacity significantly affects drug concentrations [33], while the blood–brain barrier (BBB) exhibits high selectivity, effectively separating circulating blood from brain tissue and maintaining neuronal stability and health [34,35]. These findings suggest that arecoline may be preferentially distributed through the peripheral circulation, rapidly accessing target receptors to influence neurotransmitter synthesis and reuptake. In contrast, the liver and brain maintain relatively stable neurotransmitter levels because of their efficient metabolic capacity and the isolating effect of the BBB. This difference has some guiding significance for the diagnosis of diseases and the development of drugs because the neurotransmitters in the serum are more “dynamic”, so subsequent research on neurological systems could prioritize serum-based neurotransmitter detection. However, this interpretation is speculative and should be confirmed in future studies.

### 3.2. Arecoline May Affect Mouse Behavior by Affecting Digestive Enzyme Activity

Following food ingestion, animals utilize digestion and absorption processes to acquire nutrients and energy. The intestinal microbiota, which serves as a pivotal regulator of host energy metabolism, plays essential roles in food digestion and nutrient assimilation [36,37]. The complex and diverse microbial communities in the intestinal produce various organic acids, nutrients, and enzymes that participate in critical biochemical processes and material cycling within the intestinal tract, thereby exerting significant impacts on host growth, development, and immune function [38]. Digestive enzymes, as products of the intestinal microbiota, facilitate the hydrolysis of macromolecular nutrients into absorbable small molecules, playing a crucial role in gastrointestinal physiology [39]. Moreover, supplementary enzymes can treat many intestinal diseases, so the detection of enzyme activity in the intestines can also be used to assess the effects of drugs on the body [40,41]. Under normal physiological conditions, energy intake and expenditure remain balanced. When the body’s energy intake increases, energy consumption also increases to maintain energy homeostasis [42]. Studies have shown a certain correlation between energy imbalance and neuronal excitability [43]. Therefore, the ability of arecoline to regulate digestive enzyme activity may partially mediate its stimulatory effects by altering the digestion and absorption efficiency of energy substrates, thereby promoting a short-term energy supply. Our study revealed that, compared with those in the normal group, arecoline reduced protease activity while increasing lactase and amylase activities, thereby altering the capacity of the mice to digest and absorb different nutrients. Studies indicate a correlation between sympathetic nervous system dysfunction and amylase activity, with healthy individuals exhibiting higher amylase levels than depression patients [44]. In this study, arecoline increased amylase activity, potentially accelerating carbohydrate breakdown to elevate blood glucose levels and provide short-term energy bursts for murine activity. The observed reduction in protease activity may decrease tryptophan absorption, consequently lowering 5-HT levels [45]. Research has demonstrated that 5-HT strongly inhibits the spontaneous motor behavior of mice in the open field test in a short period of time [46]. Therefore, a decrease in protease activity can relieve the inhibition of motility by 5-HT. Lactase hydrolyzes lactose into glucose and galactose, which are readily absorbed by the human body [47]. These monosaccharides serve as crucial energy substrates for systemic metabolism and essential structural sugars for metabolism in the brain and mucosal tissue. Galactose and glucose constitute key components of 2′-fucosyllactose (2′-FL), a bioactive compound that has significant immunomodulatory and anti-inflammatory properties. 2′-FL has been shown to suppress neuronal apoptosis in aged murine brains, exerting neuroprotective effects [48]. Therefore, the specific regulation of digestive enzymes by arecoline leads to increased short-term energy supply and altered neurotransmitter balance, thereby providing the material basis for increased locomotor duration in mice. This may be one of the mechanisms underlying the stimulant effects of arecoline.

### 3.3. Arecoline May Impact Host Health Through the Modulation of the Intestinal Microbiota

Sequencing analysis of small intestinal contents revealed that arecoline administration significantly altered the microbial community structure. Beta diversity analysis demonstrated that compared with the normal group, arecoline not only increased microbial species diversity but also increased interindividual variability in the composition of the intestinal microbiota. ROC analysis showed that *Escherichia* and *Clostridium* are key candidates for investigating the effects of colines on the intestinal microbiota. LEfSe analysis further identified *Escherichia* as a significantly enriched genus in the arecoline group compared with the normal group. *Escherichia*, a normal commensal bacterium in the intestinal tract of humans and animals, remains harmless under balanced intestinal microecological conditions. However, when intestinal microecological homeostasis is disrupted, an overgrowth of enteropathogenic *Escherichia coli* (EPEC) may occur, potentially inducing various diseases and even triggering epidemic infections [49,50]. Michaela J. Day et al. reported that *Escherichia* coli is the most prevalent bloodstream pathogen in England and is capable of producing extended-spectrum β-lactamases (ESBLs) and causing bacteremia [51]. Studies have demonstrated that diarrhea remains a leading cause of mortality in children under 5 years old, with diarrheagenic Escherichia strains playing a major etiological role. Notably, *Escherichia albertii* has been identified as the primary causative agent of infantile diarrhea in Vietnam [52]. However, in this study, 16S rRNA sequencing did not permit strain-level or virulence gene identification. Therefore, although the increased abundance of *Escherichia* raises potential concerns, we cannot confirm the presence of pathogenic strains without further molecular evidence such as PCR-based virulence gene detection. Future studies should address this limitation to clarify the functional implications of *Escherichia* enrichment. Furthermore, in this study, *Clostridium* was identified as one of the top 20 characteristic genus-level bacteria with an ROC analysis AUC > 0.8, playing a key role in investigating arecoline’s effects on small intestinal microbiota in mice. *Clostridium* predominantly inhabits soil, human and animal intestines, and decomposing organic matter. While most species are non-pathogenic, a minority are pathogenic. This genus plays a crucial role in producing short-chain fatty acids (SCFAs) within the intestinal. Research has identified *Clostridium* as a promising probiotic candidate that synergizes with chitooligosaccharides (COSs) to increase the content of SCFAs in the intestine, alleviate intestinal inflammation, and restore the intestinal barrier [53]. In this study, we observed that compared with the normal group, arecoline reduced the relative abundance of *Clostridium* in mouse small intestinal contents. We preliminarily speculate that arecoline may adversely affect the survival of *Clostridium* in the intestinal tract, potentially leading to decreased SCFAs concentrations and consequent impairment of intestinal immune function. Furthermore, the enrichment of *Escherichia* poses additional challenges to the maintenance of intestinal health in mice.

### 3.4. Arecoline’s Stimulant Effects May Be Mediated by Intestinal Microbiota

**Figure 8:**
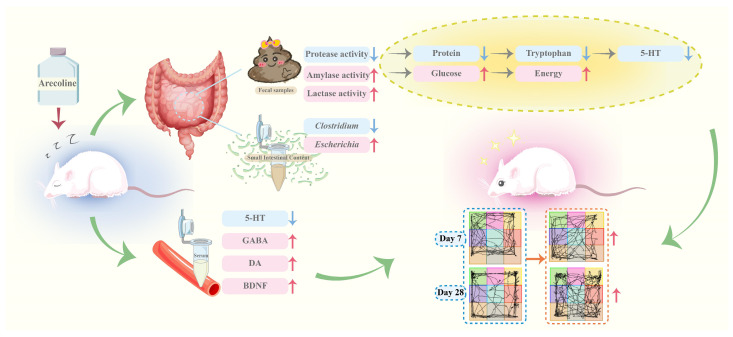
Mechanism diagram of arecoline’s stimulatory effects. The red upward arrow represents an increase, the blue downward arrow represents a decrease.

A bidirectional relationship exists among the brain, intestinal, and intestinal microbes, with substantial evidence indicating that alterations in microbial composition or abundance can influence brain health [54]. Studies have demonstrated strong correlations between the intestinal microbiota and biochemical markers such as 5-HT, NE, and serum corticosterone (CORT) in rats. Notably, yeast extract has been shown to alleviate depressive symptoms by modulating intestinal microbial communities [55]. Additional studies have shown that astragalus polysaccharides can influence intestinal SCFAs levels by modulating the abundance of the intestinal microbiota, thereby alleviating the decline of cecal SCFAs levels induced by chronic fatigue syndrome (CFS) and consequently mitigating CFS [56]. Among the causes of fatigue, lactic acid accumulation, energy depletion, and central nervous system dysfunction are associated with the intestinal microbiota, demonstrating the crucial role of intestinal microbial communities in the bioactivity of antifatigue foods [57]. Correlation analysis in this study revealed that 5-HT levels were positively correlated with *Clostridium* abundance and negatively correlated with *Escherichia* abundance, suggesting that both *Clostridium* and *Escherichia* may participate in or influence 5-HT synthesis or regulation, thereby affecting host neural function through this pathway. Conversely, GABA, DA, and BDNF levels were positively correlated with *Escherichia* abundance but negatively correlated with *Clostridium* abundance. These findings indicate that *Clostridium* may modulate host levels of these neuroactive substances by either suppressing their synthesis or promoting their degradation. While our findings indicate correlations between intestinal microbiota and neurotransmitter levels, direct causality has yet to be established. Future research employing germ-free animal models could help determine causal relationships. Moreover, targeted manipulation of specific microbial taxa or their metabolites (e.g., short-chain fatty acids) may further elucidate the mechanisms underlying microbiota–brain interactions (Figure 8).

## 4. Materials and Methods

### 4.1. Materials

#### 4.1.1. Experimental Animals and Housing Conditions

Twenty male KM mice (body weight: 18–22 g; specific pathogen-free [SPF] grade) were purchased from Hunan Silaike Jingda Laboratory Animal Co., Ltd. (Changsha, China, SCXK [Xiang] 2019-0004). All the mice were housed in the Animal Experiment Center of Hunan University of Chinese Medicine (SYXK [Xiang] 2019-0009). This study was ethically reviewed and approved by the Institutional Animal Care and Use Committee of Hunan University of Chinese Medicine, under approval number HNUCM-21-2312-09 (Approval Date: 8 November 2023).

#### 4.1.2. Animal Feed

The mice were fed with γ-irradiated (Co60) breeding feed for laboratory rodents provided by the Animal Experiment Center of Hunan University of Chinese Medicine and manufactured by Jiangsu Meidi Biomedical Co., Ltd. (Yangzhou, China, SYXK [Xiang] 2020-0006). This product contains ≤100 g of moisture, ≥200 g of crude protein, ≥40 g of crude fiber, ≤50 g of crude fat, ≤80 g of crude ash, 10–18 g of calcium, 6–12 g of phosphorus, a calcium–phosphorus ratio of 1.2:1–1.7:1, ≥13.2 g of lysine, and a total of ≥7.8 g of methionine and cysteine.

#### 4.1.3. Drugs and Kits

Arecoline (C_8_H_13_NO_2_, S26HB195898, HPLC ≥ 98%, Store at 2–8°) was purchased from Shanghai Yuanye Bio-Technology Co., Ltd. (Shanghai, China). GABA, DA, 5-HT, and BDNF (JM-02725M2, JM-02906M2, JM-02726M2, and JM-02487M2, respectively) ELISA kits were purchased from Jiangsu Jingmei Biotechnology Co., Ltd. (Yancheng, China).

### 4.2. Methods

#### 4.2.1. Animal Grouping and Drug Administration

After 3D adaptive feeding, the mice were randomly divided into a normal group and an arecoline group, with 10 mice in each group. The dosage of arecoline was based on the average daily arecoline intake of adults and controlled within the acute toxicity dose range. The arecoline group mice were administered 2 mg/(kg·d) arecoline solution by gavage, twice a day, 0.3 mL each time, for 30 consecutive days. At the same time, the normal group was given an equal amount of sterile water by gavage [16].

#### 4.2.2. Open Field Test

On the 7th, 14th, 21st, and 28th days of the experiment, an open field test was conducted. An open field box with a cylindrical shape and a bottom evenly divided into 16 square regions was used. The mouse was placed in the center of the bottom of the open field box, and after adapting for 1 min, the activity–time (%), time spent in the central area (S), and distance traveled in the central area (cm) of the mouse within 6 min were recorded via the SMART real-time video image tracking and analysis system (KSYY-OP-V4.0, Beijing Zhongshi Dichuang Technology Development Co., Ltd., Beijing, China). The experiment was conducted in a dimly lit, quiet environment [58].

#### 4.2.3. Enzyme Activity Assay

Fresh fecal samples were collected every 10 days throughout the experimental period for enzyme activity analysis. Add the sterile water to samples proportionally (sample–sterile water = 3 g:50 mL), shake for 30 min, centrifuge at 3000 r/min for 15 min, and collect the supernatant as crude enzyme extract (High-speed refrigerated centrifuge, 5425, Eppendorf SE, Hamburg, Germany). Measure the enzyme activity in the crude enzyme extract using a UV-visible spectrophotometer (722, Shanghai Sunny Hengping Scientific Instrument Co., Ltd., Shanghai, China). Amylase is measured at 520 nm using the DNS method; sucrase is measured at 540 nm using the DNS method; protease is measured at 660 nm using the Folin phenol method; and lactase is measured at 420 nm using the ONPG method [59,60].

#### 4.2.4. Detection of GABA, DA, 5-HT, and BDNF in Serum

Blood samples were collected from all groups into 1.5 mL EP tubes and left at room temperature. Following centrifugation at 3000 rpm for 10 min, the supernatant serum was carefully aliquoted for subsequent analysis. Follow the instructions on the kit to add reagents, incubate, wash the plate, and detect. The Optical Density (OD) values of each well was detected by a multimode microplate reader (SPARK, Tecan Schweiz AG, Männedorf Switzerland). Calculate the concentrations of GABA, DA, 5-HT, and BDNF in mouse serum based on the OD values [61].

#### 4.2.5. DNA Extraction, 16S rRNA Gene Amplicon Sequencing, and Sequence Analysis

① Sample collection: In a sterile environment, the contents of the mouse small intestine were collected, placed in a 1.5 mL EP tube, and labeled. The samples were flash-frozen in liquid nitrogen and subsequently stored at −80 °C in designated groups until further processing [62].

② Microbial total DNA extraction and detection: After pretreatment of the sample, nucleic acids were extracted via the OMEGA Soil DNA Kit (D5635-02; Omega Bio-Tek, Norcross, GA, USA). Molecular size determination of the extracted DNA was performed via 0.8% agarose gel electrophoresis, and DNA quantification was carried out via Nanodrop (Thermo Scientific, Waltham, MA, USA, NC2000).

③ PCR amplification and product purification: PCR amplification was performed via the following bacterial 16S rRNA V3-V4 region-specific primers: forward primer 338F (5′-ACTCCTACGGGAGGCAGCA-3′) and reverse primer 806R (5′-GGACTACHVGGGTWTCTAAT-3′). The PCR products were analyzed via 2% agarose gel electrophoresis and purified via the Axygen Gel Extraction Kit.

④ Quantification of PCR products: The Quant-iT PicoGreen dsDNA Assay Kit (Invitrogen, Waltham, MA, USA, P7589) was used on a microplate reader (BioTek, Winooski, VT, USA, FLX800T) to quantify the recovered PCR amplification products. On the basis of the required sequencing data volume for each sample, the samples were mixed proportionally.

⑤ Library Preparation and Sequencing: An Illumina TruSeq Nano DNA LT Library Prep Kit (San Diego, CA, USA) was used for library construction. After quality control and quantification of the libraries, the qualified libraries were sequenced on either the Illumina NovaSeq (PE250 paired-end sequencing) or Illumina MiSeq (PE300 paired-end sequencing) platform. The sequencing work was performed by Shanghai Personal Biotechnology Co., Ltd., Shanghai, China. The sequencing data of the small intestinal content microbiota have been deposited in the NCBI database under accession number PRJNA1121031.

#### 4.2.6. Bioinformatics Analysis

① Species Annotation: Raw data were obtained via paired-end sequencing of community DNA fragments via the Illumina platform. The primer sequences were trimmed using QIIME cutadapt trim-pairs, and sequences without matched primers were discarded. Then, quality control, denoising, assembly, and chimera removal were performed through QIIME DADA2 denoise-paired calling DADA2 for data processing. The obtained ASV feature sequences were compared with database reference sequences to obtain the corresponding taxonomic information for each ASV. With the use of QIIME 2 software (https://github.com/QIIME2/q2-feature-classifier, accessed on 13 April 2025), sequence numbers were randomly extracted from the ASV abundance matrix of each sample at different depths, and species annotation was performed via the naive Bayes classifier. Rarefaction curves were plotted using the extracted sequence numbers and corresponding ASV numbers at each depth, and the quality of the sequencing data was evaluated on the basis of rarefaction curves and abundance rank curves [63].

② Alpha diversity analysis: Alpha diversity refers to indicators of species richness and diversity in locally uniform habitats. Using QIIME 2 software, the Chao1 index, observed species index, Shannon index and Simpson index of each sample were calculated to compare the richness and evenness of ASVs among different samples [64].

③ Beta diversity index analysis: The beta diversity index reflects differences in microbial communities between samples. Dimensionality reduction was performed through principal coordinate analysis (PCoA) and nonmetric multidimensional scaling (NMDS) to demonstrate the main trends of data variation [65].

④ Species difference and signature species analysis: The VennDiagram package and R scripts were used to generate petal diagrams or Venn diagrams to visualize shared and unique species among samples. Composition and abundance tables at different taxonomic levels were obtained via QIIME2 software and are presented as bar plots. Linear discriminant analysis (LDA) combined with effect size measurements (LEfSe) was employed to detect differentially abundant taxa between groups. Random forest analysis was performed on samples from different groups via the default settings of QIIME2 [66].

⑤ Correlation analysis: Spearman correlation coefficients were calculated between characteristic bacteria and GABA, DA, 5-HT and BDNF levels. Redundancy analysis (RDA) was applied to investigate the interaction between arecoline-induced characteristic intestinal microbiota and environmental factors in mouse intestinal contents [67].

#### 4.2.7. Statistical Analysis

Statistical analysis was performed using IBM SPSS Statistics version 25.0 (IBM Corp., Armonk, NY, USA). For normally distributed measurement data, an independent samples *t*-test was used for comparisons between two groups; for nonnormally distributed data, nonparametric tests were employed. A *p*-Value < 0.05 was considered statistically significant.

## 5. Conclusions

The results demonstrated that arecoline administration stimulated exploratory behavior and spontaneous activity in mice, significantly decreased protease activity while increasing amylase activity in the intestinal contents, and moderately altered the microbiota structure in the small intestine. *Escherichia* and *Clostridium* were identified as key taxa for investigating the impact of arecoline on the intestinal microbiota. The changes in their relative abundances were correlated with the levels of 5-HT, GABA, DA, and BDNF, which may constitute a critical mechanism underlying the stimulatory effects of arecoline. However, the influence of arecoline on intestinal microbiota is a long-term effect, and further in-depth studies are required to more clearly elucidate the balance between its potential benefits and adverse effects on the organism.

## Figures and Tables

**Table 1:** Statistical table of spontaneous activity in mice.

		Day 7	Day 14	Day 21	Day 28
**Activity time (%)**	CC	53.12 ± 9.38	50.86 ± 3.75	47.26 ± 10.18	39.30 ± 6.91
	CB	60.72 ± 7.41	55.30 ± 4.66	49.53 ± 11.56	61.85 ± 14.56 *
**Time spent in center zone (S)**	CC	18.84 ± 7.13	6.80 ± 3.80	13.20 ± 8.88	8.51 ± 6.14
	CB	23.60 ± 6.40	10.68 ± 9.22	14.00 ± 7.06	12.55 ± 6.57
**Center zone distance traveled (cm)**	CC	182.37 ± 61.87	87.03 ± 57.18	127.54 ± 45.38	78.71 ± 44.17
	CB	174.44 ± 46.96	48.57 ± 25.95	142.82 ± 21.21	111.25 ± 76.79

Note: Values are expressed as the mean ± standard deviation (n = 5). * *p* < 0.05. CC: normal group; CB: arecoline group. The figure below is the same.

## Data Availability

The data underlying this study are available within the manuscript. The intestinal content microbiota sequencing data have been uploaded to the NCBI database (https://www.ncbi.nlm.nih.gov/), NO. PRJNA1121031.
